# A single-arm pilot study: can a parental sleep intervention for sleep-disturbed young children in individual settings improve children’s sleep, crying, eating, and parental distress in mothers and fathers?

**DOI:** 10.1186/s12887-022-03631-5

**Published:** 2022-10-07

**Authors:** Marisa Schnatschmidt, Friederike Lollies, Angelika A. Schlarb

**Affiliations:** Faculty of Psychology and Sports Science, Department of Psychology, Clinical Psychology and Psychotherapy of Childhood and Adolescence, Bielefeld University, P.O.P. 10 01 31, DE-33501 Bielefeld, Germany

**Keywords:** Sleep disturbance, Early regulatory problems, Early childhood, Maternal stress, Paternal stress

## Abstract

**Background:**

Early sleep problems co-occur with crying, eating problems, and parental distress. This study investigates the impact of a parent-focused intervention to improve child sleep with the following aims: (1) To assess the impact on child sleep (sleep onset latency, frequency and duration of nighttime awakenings, frequency of bed-sharing, and nighttime food intake, total nighttime sleep duration, and sleep efficiency), child crying (frequency of crying episodes, of unexplained and unsoothable crying and of crying out of defiance), child eating difficulties, and parental distress of mothers and fathers. (2) To assess the maintenance of any changes in these areas longitudinally, at 3-month, 6-month, and 12-month follow-ups. (3) To explore at the within-subjects level, how children’s sleep, crying, eating, and parental distress changed together across all study measurement points.

**Methods:**

In this single-arm pilot study, the parents of 60 children participated in six individual sessions of a parent-focused multimodal age-adjusted cognitive-behavioral intervention to improve child sleep. Parents of 39 children (46% girls, age in months M = 22.41, SD = 12.43) completed pre- and at least one measure after the intervention. Sleep diary, questionnaire for crying, feeding, sleeping, and parental stress index (short-form) were assessed pre, post, three, six, and 12 months after the intervention.

**Results:**

Significantly, sleep (decreased sleep onset latency, frequency, duration of nighttime awakenings, bed-sharing, nighttime food intake; increased total nighttime sleep duration, sleep efficiency), crying (reduced frequency of crying episodes, unexplained and unsoothable crying), and parental distress (reduced) changed, which remained partially stable over follow-up. The frequency of crying episodes decreased with fewer nighttime awakenings; morning crying with increased nighttime feeding; unexplained and unsoothable crying with higher sleep efficiency; crying due to defiance with more nighttime awakenings, sleep efficiency, and bed-sharing. Eating problems decreased with shorter night awakenings and time; maternal distress with fewer nighttime awakenings, paternal with less child’s nighttime feeding, unexplained and unsoothable crying, and time.

**Conclusions:**

A parental sleep intervention for sleep-disturbed young children could be promising to reduce children’s sleep problems, crying, eating problems and parental distress. Future studies should consider more personal contact during the follow-up to reduce the drop-out rate and a randomized-controlled design.

**Trial registration:**

The study was retrospectively registered at the German Clinical Trials Register (ID: DRKS00028578, registration date: 21.03.2022).

**Supplementary Information:**

The online version contains supplementary material available at 10.1186/s12887-022-03631-5.

## Background

Learning to self-regulate is an essential transactional task between child and caregivers in early childhood, with sleep playing a crucial role in this process [1–5]. Early regulatory problems manifest in a triad of symptoms with child behavior problems and severe parental distress and a dysfunctional parent-child interaction [5]. Child behavior problems in early regulatory problems include sleep, crying, and feeding or eating difficulties [6]. A meta-analysis including 22 studies mainly from North America, Europe, and Australia, postulated that about 20% of infants are affected in the first year of life [7]. They have been associated with various short- and long-term consequences, including impaired self-regulating, social skills, mental health, and parent-child relationships [7–12]. In addition, early regulatory problems were discussed as the earliest indicators of mental health problems in childhood [13]. Regulatory problems in sleeping, crying, and feeding or eating often co-occur and are interconnected with each other following the triad of symptoms [ 5, 6, 14]. For example, sleep problems often involve increased crying in the evening because the child has impaired emotion regulation due to fatigue or expresses protest against going to bed by crying [1, 5, 6, 15, 16]. Parents often offer food in the form of breastfeeding or bottles as a calming strategy and help to fall asleep, but this makes it more difficult for the child to learn how to fall asleep independently as well as a positive daytime eating routine [17]. The already increased distress on the parents in the context of a child’s sleeping problems can also increase due to further regulation difficulties, which is expressed in a more tense parent-child interaction, which intensifies the child’s problem behavior [5]. Sleep, crying, feeding, or eating problems have in common that the child’s self-regulatory competence is not yet sufficiently developed, the parents are overwhelmed and severely stressed, and the parent-child interaction is impaired [5]. But adequate parental co-regulation in a supportive parent-child interaction is essential to overcome the child’s regulatory problems [5]. Thus, if the parent-child system is burdened by one regulation problem, it is plausible that child regulation behavior in other domains will also be impaired [1, 3, 5, 18]. In a German study, multiple regulation disorders affected about 3.4% of children at 12 months of age [19]. In an Australian longitudinal study of 376 children between two and 24 months of age, 25% of parents reported cry-fuss/sleep problems combined at least once, while they persisted in 6% over at least three-time points [20]. In infancy as well as in toddler and preschool years, sleep problems are associated with short- and long-term impaired regulatory capacity, which is associated with crying [4, 15, 21–26].

The prevalence of early childhood sleep problems, defined as difficulty falling and staying asleep, difficulty sleeping alone, and bedtime resistance, ranges from 10 to 40% [20, 27–29]. The high effectiveness of behavioral interventions for sleep problems in early childhood in improving children’s sleep has been shown in intervention studies as well as in systematic reviews and meta-analyses of controlled trials and within-subject studies [30–33]. However, to date, no study has shed light on whether sleep interventions may also improve crying and feeding or eating problems.

Childhood crying problems involve unexplainable, unsoothable crying, and in toddlers often goes along with increased crying due to defiance [6, 34]. Childhood crying problems are often accompanied by high levels of parental distress [5, 6, 34]. In children between 6 and 47 months, the prevalence of persistent excessive crying is 2.5% [14]. When parent-reported mild or moderate crying in infancy occurs together with other regulatory problems, the risk of long-term mental health problems is increased [19]. In toddler and preschool age, the relationship between sleep problems and crying is influenced by the development of the child’s autonomy, resulting in more crying due to defiance, especially as resistance to bedtime, but also due to tiredness during the day [15, 16, 21, 24, 26, 34].

Feeding or eating difficulties include picky eating behavior, reluctance or refusal to eat, interaction at mealtimes perceived as problematic by parents, and single meals lasting more than 45 minutes [17, 34]. The prevalence is about 1.4% in the first year and about 3% in the age from 13 to 48 months [14].

Parental distress describes a parent’s perception of stress about performing the parenting role [35]. According to the triad of symptoms, parental distress is essential to early childhood regulatory disorders [5]. Various studies showed associations of increased parental distress with child sleep problems [36, 37], crying problems [20, 36], and feeding or eating problems [36, 38], as well as with the co-occurrence of these regulatory problems [10, 37, 39–41]. Although many studies have focused on maternal distress, there is also evidence that fathers are burdened by a child’s sleep and crying problems [37, 41].

Different mechanisms could influence children’s sleep, crying, eating behaviors, and parental distress. Since the present study focused on the context of a parent-centered intervention to improve a child’s sleep, several mechanisms are possible. Greater parental knowledge about child sleep and fewer dysfunctional parental cognitions about child sleep behavior are associated with more positive sleep-related parental behaviors, such as good sleep hygiene for the child which is related to improved child sleep [42]. Since individual parenting strategies for challenging situations particularly include children’s crying episodes in the context of sleep problems, it could be assumed that child’s crying problems may also be reduced if parenting strategies are taught to parents [32]. A child’s eating problems may decrease with improved sleep hygiene because that includes no longer child’s food intake in the evening and at night as a soothing strategy [43]. Further, improving infant sleep and regulation problems can lead to a relaxation in parent-child interaction, which is central to a positive eating routine [5]. In addition, healthy sleep behavior is associated with better emotion and self-regulation in young children which may positively impact crying and eating problems [3]. Parental distress could decrease through relaxation techniques for parents and a reduction in parental dysfunctional cognitions [43, 44]. Similarly, improved child sleep and regulation problems could relieve the parental burden and thus contribute to reduced parental distress [5]. Overall, an interaction between reduced parental distress and reduced child behavior problems may positively impact parent-child interactions, which are central to ameliorating a child’s sleep and regulation problems [5].

In addition, parents of young children consult professional help mostly due to sleep, crying, and feeding or eating problems [45, 46]. As the co-occurrence of sleep, crying, and eating problems are highly distressing for parents [10, 39–41], it is also a risk factor for child endangerment, such as shaken baby syndrome [47, 48]. In addition, the co-occurrence of regulatory problems increases the likelihood of their persistence [18, 49]. Therefore, early detection and treatment of crying and eating problems in young children affected by sleep disturbances are essential.

The purpose of this study is to investigate the impact of a parent-focused intervention to improve child sleep in individual settings with parents of young children with disturbed sleep. Therefore this study has three main aims: Aim (1) is to assess the impact of the parent-focused intervention to improve child sleep on child sleep (sleep onset latency, frequency and duration of nighttime awakenings, frequency of bed-sharing, and nighttime food intake, total nighttime sleep duration, and sleep efficiency), child crying (frequency of crying episodes, of unexplained and unsoothable crying and of crying out of defiance), child eating difficulties, and parental distress of mothers and fathers. Aim (2) is to assess the maintenance of any changes in these areas longitudinally, at 3-month, 6-month, and 12-month follow-ups. Aim (3) is to explore at the within-subjects level, how children’s sleep, crying, eating, and parental distress changed together across all study measurement points.

## Methods

### Participants and procedure

The parents of 60 children participated in the study, including three sets of two-parent families with two children and six children being raised by single mothers. Table 1 provides the demographic characteristics of the sample. Families were recruited primarily through information brochures in pediatric practices, the psychotherapeutic university outpatient clinic for children and adolescents, counseling centers, parenting classes, and kindergartens in the local area of Bielefeld (North Rhine-Westphalia, Germany). In addition, we published a press release in the local newspaper and held information sessions for local professionals to support recruitment. Participation was free of charge. Inclusion criteria were the children’s age between 6 months and 4 years, behavioral sleep problems (for at least 4 weeks), and the completed parental consent form. High levels of crying, eating problems, and parental distress were not inclusion criteria. The International Classification of Sleep Disorders (3rd edition) [50] and age-related criteria [51], adjusted to ages between 6 and 48 months, determined the sleep disturbance diagnostic as indicated by the Mini-KiSS Anamnestic Questionnaire, the German version of the Children’s Sleep Questionnaire [52], and diagnostic interviews by trained psychologists and child and adolescent psychotherapists in professional education. The same materials (see instruments) were completed at a total of five measurement time points (T1: before, T2: after, T3: 3, T4: 6, and T5: 12 months after the intervention). The study complied with the Declaration of Helsinki and was reviewed and approved by the Ethics Committee of Bielefeld University (Nr. 2017–036). The study was retrospectively registered at the German Clinical Trials Register (ID: DRKS00028578, UTN: U1111–1275-7616).

**Table 1:** Sample characteristics and attrition analysis

Sample characteristics	Total	Drop-out	Non-dropout	Drop-out vs. Non-dropout
Children	*N* = 60	*n* = 21	*n* = 39	
female child, n (%)	30 (50)	12 (57.14)	18 (46.15)	*χ2* (1) = 0.66, *p* = 0.417
age, months, *Mdn* (*Q1-Q3*)	17 (12.00–26.75)	15 (12.00–26.50)	18 (13.00–34.00)	*z* = 0.67, *p* = 0.505
Mothers	*N* = 57	*n* = 20	*n* = 37	
age, years *Mdn* (*Q1-Q3*)	34.00 (31.25–36.00)	33.00 (30.25–35.00)	35.00 (32.00–36.00)	*z* = 1.13, *p* = 0.260
high-school or higher education, n (%)	24 (42.85)	9 (45.00)	15 (41.66)	*χ2* (1) = 0.058, *p* = 0.809
employed (full- or part-time), n (%)	31 (58.49)	9 (45.00)	22 (61.11)	*χ2* (1) = 0.317, *p* = 0.573
Fathers	*N* = 51	*n* = 18	*n* = 33	
age, years, *Mdn* (*Q1-Q3*)	36.00 (32.00–39.00)	34.00 (30.75–39.00)	36.50 (34.00–39.00)	*z* = 1.58, *p* = 0.114
high-school or higher education, n (%)	25 (50.00)	10 (55.55)	15 (46.87)	*χ2* (1) = 0.347, *p* = 0.556
employed (full- or part-time), n (%)	46 (97.87)	15 (93.75)	31 (100.00)	*χ2* (1) = 1.980, *p* = 0.159

‘Drop-out’ refers to participants who dropped out after T1. ‘Non-dropout’ refers to participants who completed at least T1 and one additional measurement point. Chi-square and Mann-Whiney *U* tests were used to test differences between drop-outs and non-dropouts. *Z* stands for the *z*-scores of the Mann-Whiney *U* tests. The percentage value refers to the proportion of available data

*Abbreviations*: *Mnd* Median, *Q1* 25th percentile, *Q3* 75th percentile

**Fig. 1:**
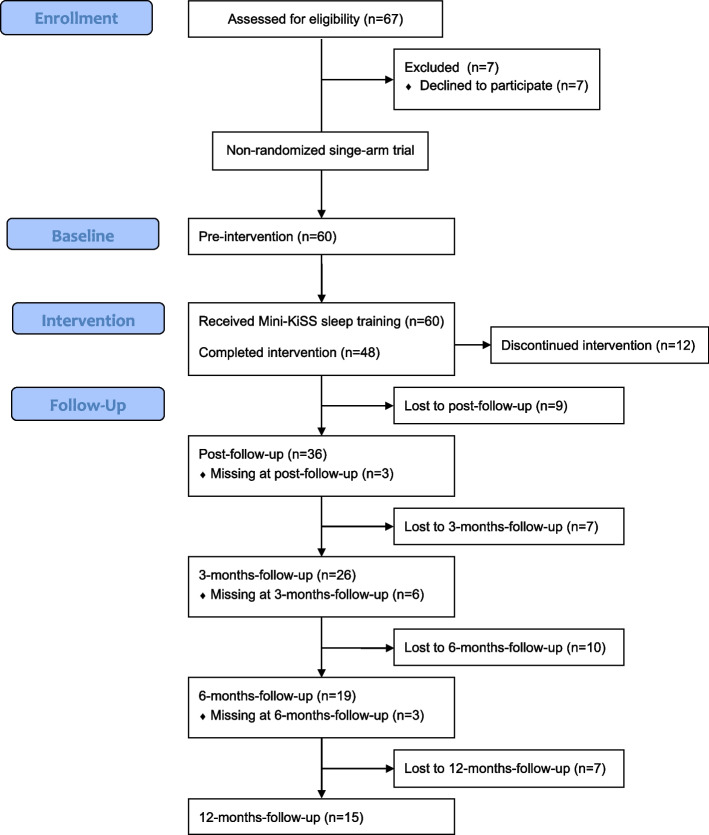
Flow diagram of the study participants. “Missing data” refers to data from children who were missing at the corresponding measurement time point but were not completely dropped out of the study because their data were available at a later measurement time point

Figure 1 provides the process of participation. Reasons for families to discontinue the study were mainly related to an overload of stress in daily family life, work, or a move. A drop-out analysis focusing on sample characteristics is presented in Table 1.

### Intervention

The Mini-KiSS sleep training is a multimodal parent training program based on age-adapted cognitive behavioral therapy and includes six treatment sessions. All sessions were conducted under the supervision of the intervention’s developer, Prof. Schlarb, at the Psychotherapeutic University Outpatient Clinic for Children, Adolescents and their Families in Bielefeld, Germany. The two main trainers were clinical psychologists, supported by five child and adolescent psychotherapists in training. All interventionists completed training on conducting the Mini-KiSS sleep training before working with the families. All sessions were conducted exclusively with the parents. In this study, sessions were conducted individually with one parent pair in a face-to-face setting once a week for 6 weeks. The main components of the intervention involved a) parental knowledge about children’s healthy sleep and sleep disturbances in childhood, b) individually sleep-related strategies for improving sleep hygiene and parenting skills, c) relaxation techniques for parents and children, and d) reduction of dysfunctional parental cognitions. More details of the intervention are described in the therapy manual by Schlarb [43]. A detailed overview of the Mini-KiSS sessions, contents, and methods is also provided in an article by Schlarb and Brandhorst [53]. Mini-KiSS sleep training was shown to improve children’s sleep behavior [33, 53]. However, this is the first study that implements the Mini-KiSS sleep training in individual instead of group or online settings.

### Instruments

#### Sleep dairy

Parents completed a sleep diary based on Schlarb and colleague’s work [33], reporting their child’s sleep over 14 days at each measurement time-point (T1: before, T2: after, T3: 3, T4: 6, and T5: 12 months after the intervention) to assess sleep onset latency (SOL), frequency of nightly awakening (FNW), duration of nightly awakening (DNW), bed-sharing (BS), nightly food intake (NFI), and general information on the child’s sleep (total nightly sleep duration (TSD), sleep efficiency (SE: ratio of minutes of sleep compared to the number of minutes in bed)). The first week was for adaptation, and only the second week was included in the analysis. Moreover, special events were documented. In the present study, sleep problems were defined according to Gaylor [51] with the following criteria: (1) Sleep disturbance (at least 2 episodes per week with at least 2 of 3 criteria met): a) Sleep onset latency > 30 minutes (between 12 and 23 months) or sleep onset latency > 20 minutes (older than 24 months), b) child needs a parent in the room to sleep, c) child struggles with bedtime; (2) night waking disturbance (at least 2 episodes per week): nighttime awakenings ≥2 (between 12 and 23 months) or nighttime awakenings ≥1 (older than 24 months), and a total of ≥20 minutes awake during the night.

#### Questionnaire for crying, feeding, and sleeping

Crying was assessed with seven items from a standardized questionnaire titled the SFS (which stands for “Schreien, Füttern, und Schlafen” in German, and translates to “Crying, Feeding, and Sleeping” in English [54]. Parents answered questions on a four-point scale (“1 = never/seldom”, “2=sometimes/1-3 per week”, “3=often/4-6 times per week, “4 = always/daily“) that related to a typical week. The SFS had high internal consistency (*α*=0.8-0.9).^46^ However, because the SFS was developed for the first year of life^46^ and the present sample includes children up to four years of age, a principal component analysis was conducted with the seven cry items in the current sample. A three-factor solution was found. The first factor describes the frequency of crying (FRQ, items 1-4: crying in the morning, afternoon, evening, and night), while the second factor describes the concept of unexplained and unsoothable crying (UUC, items 5-6: Parents do not see any reason for the crying, parental attempts to soothe the child cannot reduce the crying), and the third factor describes crying out of defiance (DEF, item 7). The difficulty of the feeding or eating patterns of the child was assessed with the feeding subscale of the SFS, which consists of a total of 13 items, and high values indicate more difficulties. We refer to feeding or eating problems as ‘eating problems’ due to the children’s age up to four years included. The internal consistency in the present sample was acceptable to good for the FRQ scale (T1: *α*=0.72, T2: *α*=0.82, T3: *α*=0.84, T4: *α*=0.85, T5: *α*=0.74) and the feeding subscale (T1: *α*=0.77, T2: *α*=0.80, T3: *α*=0.72, T4: *α*=0.87, T5: *α*=0. 74). The UUC scale was low at T1 and T2 but could be rated as good at the last three measurement time points (T1: *α*=0.53, T2: *α*=0.59, T3: *α*=0.84, T4: *α*=0.80, T5: *α*=0.80). There are no cut-off values for crying or eating problems for the SFS, and a diagnosis of clinical feeding or eating problems cannot be determined based on the SFS. However, we define problematic crying and eating patterns perceived by the parents with the SFS Items: “How much do you feel bothered by your child’s crying and whining/ eating patterns? How much do you find it problematic?“. The answers “not at all“ indicated no problem, and “mild”, “moderate“, or “severe” indicated problematic crying or eating.

#### Parental stress index short form

The German version of the Parental Stress Index Short Form (PSI-SF) [35] evaluated the parental distress. This short-form includes 36 statements about parental perceptions in general, answered on a five-point scale, with response options from “strongly agree” to “do not agree at all”, with high values indicating high parental distress. The PSI-SF was given separately to mothers and fathers. In the present analyses, only the subscale parental distress was used, which includes 12 items and was shown to have good internal consistency in previous research (*α* = 0.87) [35] as well as in the present sample (mothers: *α* = 0.78–0.91; fathers: *α* = 0.83–0.95). High levels of parental distress were, according to Abidin [35], above the 85th percentile.

### Statistical analyses

The Statistical Package for the Social Sciences SPSS 27.0 for Windows was used for the analyses [55]. To analyze differences between drop-outs (after T1) and non-dropouts (who had completed at least T1 and another measurement time point), the sample demographics at baseline were analyzed using chi-square, Mann-Whiney *U* tests. Due to the non-normal distribution, Kruskal-Wallis and Mann-Whiney *U* tests were calculated to assess significant differences in sleeping parameters, crying, eating, and parental distress scales at baseline (T1) between a) the child’s initial age in the first, second, third, or fourth year of life, b) female and male children, and c) study drop-out.

To examine the difference between T1 and the following measurement time points (T2, T3, T4, T5) in sleep, cry, eat parameters, and parental distress, Wilcoxon signed-rank tests were calculated. Since these analyses were based on one subject’s data at two timepoints as matched pairs, they mitigated the different sample sizes at each measurement point [56]. To explore, how children’s sleep, crying, eating, and parental distress changed together within a child across all study measurement points, linear mixed models (LMMs) were the method of choice due to the nested data structure and their robustness in missing data. Recent research has shown that LMMs are robust to violations of distributional assumptions [57], so we use LMMs here even though our data are not normally distributed. To explore how children’s sleep, crying and eating change together within a child across all measurement points, LMMs were calculated for crying and eating variables separately, with the sleep variables as fixed effects. To explore how children’s sleep, crying, eating, and parental distress change together within a child across all measurement points, LMMs were calculated for parental distress of mothers and fathers separately, with the child’s sleep, crying, and eating variables as fixed effects. Each model’s predictor variables (sleep, crying, and eating parameters) were at the within-subject level. For the crying and the eating scales as outcomes, the seven sleep variables were fixed effects, and for the parental distress scales as outcomes, the seven sleep variables, the three crying scales, and the eating scale were fixed effects. Every model controlled the within-subject covariate time and the between-subject covariate initial age and sex. The multivariate approach considers the influence of the other sleep, crying, and eating parameters. All models were specified with a random intercept, the maximum likelihood estimation procedure, the diagonal covariance structure, and the Satterthwaite adjustment. All predictor variables were grand mean-centered, and all models were built with two levels (Level 2: individuals, Level 1: measurement points). According to the recommendation of Nezlek [58], the models were built due to the forward-stepping strategy. Therefore, the final models included only significant variables, indicating that these significant variables change together with the outcome variable within one child across all measurement points. Due to the pilot nature of the study, no *p*-value adjustment was applied in this procedure. Available case analyses were performed with varying sample sizes due to missing values (see Twisk et al.) [59].

For the analyses of the differences between the measurement points, the effect-sizes are given as Pearson’s correlation coefficient with *r* = I0.1I; I0.3I; I0.5I describes small, medium, and large effects [60]. For the LMM analyses, the effect sizes are given as Cohen’s *d* with *d* = |0.2|; |0.5|; |0.8| describes small, medium, and large effects [60].

## Results

The demographic sample characteristics are displayed in Table 1. Of the sample of 60 children, 21 participated only at T1, and 39 children in either the initial post measurement (T2, *n* = 36) or at least one follow-up measurement (T3-T5, n = 3). The children who dropped out after T1 and those who continued showed no significant differences in the demographics at baseline (Table 1). Mann-Whitney *U* tests showed no significant differences in the outcome variables at baseline between study drop-out (*p* > .05, see Supplementary Table 1, Additional File 1). Kruskal-Wallis tests showed significant differences in frequency of nightly awakening (*χ2*(3) = 22.7, *p* < .001) and duration of nightly awakening (*χ2* (3) = 16.43, *p* < .05) according to age group. Subsequent post hoc tests (Dunn-Bonferroni tests) showed that children between 6 and 11 months at baseline differed significantly from children between 24 and 35 months at baseline (frequency of nightly awakening: *z* = 3.82, *p* = <.001; DNW: *z* = 3.18, *p* < .01) and from children between 36 and 48 months at baseline (frequency of nightly awakening: *z* = 4.02, *p* < .001; duration of nightly awakening: *z* = 3.04, *p* < .05). However, there were no other significant differences according to children’s age group and sex (*p* > .05, Supplementary Table S1).

### Children’s sleep, crying and eating patterns after the sleep intervention

Table 2 displays the descriptive data for the outcomes overall five measurement points.

**Table 2:** Descriptive statistics through all five measurement points

	T1				T2			T3			T4			T5		
	*N*	*Mdn (Q1-Q3)*			*N*	*Mdn (Q1-Q3)*		*N*	*Mdn (Q1-Q3)*		*N*	*Mdn (Q1-Q3)*		*N*	*Mdn (Q1-Q3)*	
children’s sleeping																
SOL	36	20.93 (15.78–39.82)			36	19.29 (7.86–37.68)		25	20.00 (7.50–28.93)		18	18.00 (5.32–33.75)		14	27.15 (12.18–43.57)	
FNW	36	2.43 (1.29–3.82)			36	1.07 (0.43–2.53)		25	1.14 (0.14–2.23)		18	0.50 (0.00–1.80)		14	1.00 (0.40–2.04)	
DNW	36	25.22 (9.88–51.96)			36	11.93 (2.32–21.40)		25	5.71 (0.92–26.43)		18	3.34 (0.00–18.93)		13	9.29 (3.21–16.43)	
TSD	36	625.07 (590.93–654.27)			36	641.13 (609.47–663.36)		25	632.86 (605.71–676.07)		18	642.00 (615.72–676.93)		13	648.00 (598.10–674.29)	
SE	36	91.96 (88.25–94.71)			36	94.53 (90.77–97.48)		25	95.62 (93.11–97.78)		18	95.79 (92.83–98.43)		13	94.03 (91.76–96.86)	
BS	36	0.57 (0.14–1.00)			36	0.07 (0.00–0.66)		25	0.00 (0.00–0.29)		18	0.07 (0.00–1.00)		14	0.50 (0.11–1.00)	
NFI	36	0.76 (0.29–1.00)			36	0.22 (0.00–0.86)		25	0.14 (0.00–0.91)		18	0.00 (0.00–0.57)		14	0.36 (0.11–0.61)	
children’s crying																
FRQ	35	1.75 (1.00–2.25)			35	1.25 (1.00–2.00)		24	1.00 (1.00–1.50)		19	1.00 (1.00–1.75)		14	1.50 (1.00–2.06)	
UUC	35	2.50 (2.00–2.50)			35	2.00 (1.50–2.50)		24	1.50 (1.00–2.50)		18	1.75 (1.38–2.63)		14	2.50 (2.00–3.25)	
DEF	34	2.00 (2.00–3.00)			35	2.00 (2.00–3.00)		24	2.00 (1.25–3.00)		19	2.00 (2.00–3.00)		14	2.50 (2.00–3.00)	
children’s eating																
EAT	33	1.46 (1.15–1.85)			33	1.31 (1.15–1.73)		24	1.39 (1.23–1.60)		17	1.15 (1.08–1.54)		14	1.27 (1.14–1.46)	
parental distress																
PD-M	33	27.00 (21.50–33.00)			33	23.00 (18.50–29.50)		23	23.00 (18.00–30.00)		16	24.00 (19.50–29.75)		13	21.00 (15.00–37.50)	
PD-F	27	23.00 (17.00–29.00)			28	19.00 (15.00–27.00)		19	19.00 (14.00–24.00)		14	16.50 (13.75–24.25)		11	20.00 (16.00–26.00)	
		*N*	*n*	%	*N*	*n*	%	*N*	*n*	%	*N*	*n*	%	*N*	*n*	%
Sleep disturbance		36	36	100	36	20	55.6	25	11	44.0	19	11	57.9	14	10	71.4
Crying problem		35	32	91.4	35	28	80.0	24	18	75.0	18	10	55.6	14	11	78.6
Eating problem		33	11	33.3	33	11	33.3	24	3	12.5	18	5	27.8	14	3	21.4
High PD-M		33	5	15.1	33	4	12.1	23	2	8.7	16	2	12.5	13	3	23.1
High PD-F		27	2	7.4	28	3	10.7	19	1	5.2	14	1	7.1	11	1	9.1

Medians and quartiles across all five measurement time points. The baseline data (T1) are shown for the participants who also completed the post-measurement (T2). All values in percent are adjusted to the drop-out rate. ‘sleep disturbances’ refers to children with at least sleep onset and/or night waking disturbances (> = 2 episodes per week), ‘crying problems’ and ‘eating problems’ refers to children whose parents stated their children’s crying/eating patterns at least as mild problematic, ‘high PD-M’ and ‘high-PD-F’ refers to values of the PSI-SF subscale parental distress over the 85th percentile [35]

*Abbreviations*: *Mnd* Median, *Q1* 25th percentile, *Q3* 75th percentile, *T1* Pre, *T2* Post, *T3* Three months, *T4* Six months, *T5* Twelve months follow-up, *SOL* Sleep onset latency, *FNW* Frequency of nightly awakening, *DNW* Duration of nightly awakening, *TSD* Total nightly sleep duration, *SE* Sleep efficiency, *BS* Bed-sharing, *NFI* Nightly food intake, *FRQ* Crying frequency, *UUC* Unexplained and unsoothable crying, *DEF* Crying due to defiance, *EAT* Difficulties in eating behavior, *PD-M* Parental distress of mothers, *PD-F* Parental distress of fathers

Child sleep improved after the intervention. Immediately after the intervention, sleep onset latency, frequency and duration of nightly awakening, bed-sharing, and nightly food intake significantly decreased while the total sleep duration and sleep efficiency increased significantly (Tables 2 and 3). Twelve months after the intervention, these effects remained stable for frequency and duration of nightly awakenings, nightly food intake, and sleep efficiency (Tables 2 and 3). For bed-sharing, the effect was stable until 6 months after the intervention and for the reduced sleep onset latency until 3 months after the intervention (Tables 2 and 3). The effect for reduced total nightly sleep duration was no longer significant in the follow-up period (Tables 2 and 3).

**Table 3:** Results of Wilcoxon signed-rank tests for pre-, post-, and follow-up intervention differences

	T1 – T2			T1 – T3			T1 – T4			T1 – T5		
	*n*	*z*	*r*	*n*	*z*	*r*	*n*	*z*	*r*	*n*	*z*	*r*
children’s sleeping												
SOL	36	−2.22*	0.37	25	−2.34*	0.47	18	−1.81	0.43	14	−1.35	0.36
FNW	36	−3.18**	0.53	25	−3.37**	0.67	18	−3.51***	0.83	14	−2.34*	0.63
DNW	36	−3.65***	0.61	25	−2.76**	0.55	18	−3.72***	0.88	13	−3.11**	0.86
TSD	36	2.48*	0.41	25	1.12	0.22	18	1.59	0.37	13	0.52	0.15
SE	36	3.69***	0.62	25	3.22**	0.64	18	3.42**	0.81	13	2.59*	0.72
BS	35	−2.89**	0.49	25	−3.36**	0.67	18	−2.04*	0.48	14	−0.76	0.20
NFI	36	−3.07**	0.51	25	−2.47*	0.49	18	−3.42**	0.81	14	−3.08**	0.82
children’s crying												
FRQ	35	−2.28*	0.38	24	−3.01**	0.62	19	−1.40	0.32	14	−1.30	0.35
UUC	35	−1.86*	0.31	24	−2.01*	0.41	18	−0.82	0.19	14	1.62	0.43
DEF	34	1.17	0.20	24	0.72	0.15	19	1.05	0.24	14	2.16*	0.58
children’s eating												
EAT	33	−1.65	0.29	24	−0.32	0.06	17	−2.49*	0.60	14	−1.64	0.44
parental distress												
PD-M	33	−2.95**	0.51	23	−1.57	0.33	16	−0.80	0.20	13	−0.74	0.20
PD-F	27	−1.21	0.23	18	−2.89**	0.68	14	−2.13*	0.57	11	−0.71	0.21

Pre-post and follow-up analyses of the children’s sleep, crying, eating, and parental distress parameters. *Z* stands for the *z*-scores of the Wilcoxon signed-rank tests. *P*-values are one-tailed due to the directional hypotheses and are Benjamini-Hochberg adjusted. * *p* < .05, ** *p* < .01, *** *p* < .001

*Abbreviations*: *T1* Pre, *T2* Post, *T3* Three months, *T4* Six months, *T5* Twelve months follow-up, *SOL* Sleep onset latency, *FNW* Frequency of nightly awakening, *DNW* Duration of nightly awakening, *TSD* Total nightly sleep duration, *SE* Sleep efficiency, *BS* Bed-sharing, *NFI* Nightly food intake, *FRQ* Crying frequency, *UUC* Unexplained and unsoothable crying, *DEF* Crying due to defiance, *EAT* Difficulties in eating behavior, *PD-M* Parental distress of mothers, *PD-F* Parental distress of fathers

Child crying reduced after the intervention. The frequency of children’s crying episodes and unexplained and unsoothable crying episodes showed a significant reduction after the sleep intervention until 3 months, as shown in Tables 2 and 3. The crying due to defiance did not significantly decrease after the intervention (Tables 2 and 3). Twelve months after the intervention, the significant change describes increased crying due to defiance (Tables 2 and 3). The difficulties in the child’s eating patterns seemed to reduce after the intervention (Table 2), but this effect was only significant 6 months after the intervention (Table 3).

Parental distress decreased after the intervention. Maternal distress reduced significantly after the intervention (Table 3). However, the reduction during the follow-up period was not statistically significant (Tables 2 and 3). The distress in fathers did also reduce in our sample after the intervention (Table 2), but this effect was only in the follow-up period at three and 6 months after the intervention significant (Table 3).

### Associations among children’s sleep, crying, eating, and parental distress

Table 4 and Table 5 show the results of the LMMs and Cohen’s *d* calculations. The ICC, calculated from the intercept-only models, indicated that across all measurement time points for the cry parameters between 27.3 and 28.9%, for the eating scale 70.4%, and for the parental distress scales, 70.5 and 73.3% variance were explained at the individual level. The ICC for unexplained and unsoothable crying could not be calculated validly because the final Hessian matrix was not positive definite, although all convergence criteria were met. Because this error did not occur when performing the LMMs analyses, we still calculated the LMMs for the unexplained and unsoothable crying variable. The interdependence between the seven sleep variables was examined with Spearman correlations across all five measurements (see Supplementary Table 2–6, Additional File 1).

**Table 4:** Estimates of main effects across crying and eating outcomes

	FRQ			UUC			DEF			EAT		
	*β (95% CI)*	*t (df)*	*d*	*β (95% CI)*	*t (df)*	*d*	*β (95% CI)*	*t (df)*	*d*	*β (95% CI)*	*t (df)*	*d*
children's sleeping												
SOL												
FNW	0.215 (0.126–0.303)	*t* (96.99) = 4.81***	0.98				−0.276 (− 0.419 --0.133)	*t* (65.74) = −3.86***	0.95			
DNW										0.004 (0.002–0.006)	*t* (79.63) = 3.44***	0.77
TSD												
SE				−0.056 (−0.101--0.011)	*t* (74.02) = −2.48*	0.57	− 0.083 (− 0.130--0.035)	*t* (71.46) = −3.48**	0.84			
BS							−0.789 (−1.234--0.345)	*t* (66.60) = −3.45**	0.85			
NFI	−0.174 (− 0.290--0.058)	*t* (25.09) = −3.08**	1.23									
covariates												
age												
sex												
time										−0.115 (−0.220--0.011)	*t* (24.14) = −2.27*	0.92

Estimates of main effects in the final models for the three crying and the eating variables with age, sex, and time as covariates. Due to the pilot nature of the study, no *p*-value adjustment was applied. * *p* < .05, ** *p* < .01, *** *p* < .001

*Abbreviations*: *FRQ* Crying frequency, *UUC* Unexplained and unsoothable crying, *DEF* Crying due to defiance, *EAT* Difficulties in eating behavior, *SOL* Sleep onset latency, *FNW* Frequency of nightly awakening, *DNW* Duration of nightly awakening, *TSD* Total nightly sleep duration, *SE* Sleep efficiency, *BS* Bed-sharing, *NFI* Nightly food intake; age; children’s age at baseline; sex, children’s sex; time, time since study begin

**Table 5:** Estimates of main effects across parental distress outcomes

	PD-M			PD-F		
	*β (95% CI)*	*t (df)*	*d*	*β (95% CI)*	*t (df)*	*d*
children's sleeping						
SOL						
FNW	0.892 (0.091–1.693)	*t* (94.08) = 2.21*	0.46			
DNW						
TSD						
SE						
BS						
NFI				3.301 (1.207–5.396)	*t* (54.82) = 3.16**	0.85
children's crying						
FRQ						
UUC				1.293 (0.267–2.320)	*t* (58.23) = 2.52*	0.66
DEF						
children's eating						
EAT						
covariates						
age						
sex						
time				−3.373(−5.841--0.904)	*t* (29.37) = −2.79*	1.03

Estimates of main effects in the final models for the two parental distress variables with age, sex, and time as covariates. Due to the pilot nature of the study, no *p*-value adjustment was applied. * *p* < .05, ** *p* < .01

*Abbreviations PD-M* Parental distress of mothers, *PD-F*, Parental distress of fathers, *SOL* Sleep onset latency, *FNW* Frequency of nightly awakening, *DNW* Duration of nightly awakening, *TSD* Total nightly sleep duration, *SE* Sleep efficiency, *BS* Bed-sharing, *NFI* Nightly food intake, *FRQ* Crying frequency, *UUC* Unexplained and unsoothable crying, *DEF* Crying due to defiance, *EAT* Difficulties in eating behavior; age; children’s age at baseline; sex, children’s sex; time, time since study begin

The final models are described in Tables 4 and 5. The frequency of crying episodes changed together with the frequency of nighttime awakenings and nighttime food intake. A higher frequency of crying episodes was associated with more nighttime awakenings and decreased nighttime food intake. Unexplained and unsoothable crying changed together with sleep efficiency. Increased unexplained and unsoothable crying was related to a lower child’s sleep efficiency. Crying due to defiance changed together with the frequency of nighttime awakenings, sleep efficiency, and frequency of bed-sharing. More crying due to defiance was related to fewer nighttime awakenings, lower sleep efficiency, and less bed-sharing. Eating difficulties changed together with the durations of nighttime awakening and time since the study began. More difficulties in children’s eating behavior were associated with longer durations of nighttime awakening and less time progressed in the study period. The covariates time, initial age, and sex had no association with the crying parameters, and besides the time covariate, no association with the eating parameter. Parental distress of mothers changed together with the frequency of the child’s nighttime awakenings. Increased maternal parental distress was related to more child nighttime awakenings. In contrast, parental distress of fathers changed together with the child’s nighttime food intake, unexplained and unsoothable crying, and time since the study began. More paternal parental distress was associated with increased nighttime feeding and increased unexplained and unsoothable crying. Progressing time was related to decreasing paternal distress.

## Discussion

This pilot study aimed to gain initial insights into young sleep-disturbed children’s sleep, crying and eating patterns, and parental distress in the short- and long-term following a parental sleep intervention and to examine the associations within-subjects between children’s sleep, crying, eating patterns, and parental distress. Based on the data of this pilot study, there could be a trend that children’s sleep disturbances, crying, and eating problems, as well as parental distress of mothers and fathers, reduced after the intervention. However, all results should be interpreted with caution due to the lack of a control group and the high drop-out rate.

### Children’s sleep disturbances and crying problems may improve after sleep intervention

The data of this pilot study indicate that child sleep problems improved after intervention with lasting effects for up to 12 months. From pre to post intervention, sleep onset latency decreased from a median of 20 Minutes to 19 Minutes, night waking decreased by one wakeup per night with duration decreasing from a median of 25 Minutes to 12 minutes, resulting in an increase of 16 minutes in total sleep duration and 2 % of sleep efficiency. The median of bed-sharing decreased from 57% of nights to 7 % and nightly food intake from 76% of nights to 22% of nights directly after training. These improvements from pre to post intervention were stable over the follow-up period in almost all sleep parameters. The number of children who suffered from sleep disturbances and sleep disorders decreased after the intervention. These finding are in line with previous research showing the effectiveness of behavioral interventions for sleep problems in early childhood [30–33, 53]. However, this is the first study assessing the effects of the Mini-KiSS intervention in individual instead of group settings, and sheds light on effects in a long-term follow-up period of 12 months. The study results suggest that the Mini-KiSS intervention can also be effective in an individual setting, which would allow for greater flexibility in treatment in a clinical context.

Further, the child crying reduced up to 3 months after the intervention with associations to a reduction of nighttime awakenings and increased sleep efficiency. The frequency of crying episodes decreased after the parent-focused intervention with improvements on the child’s sleep from a median of 1.75 to a median of 1.25, and the frequency of unexplained and unsoothable crying episodes from a median of 2.50 to 2.00, with a value of 1 indicating crying episodes occurred seldom to never and a value of 2 indicating an occurrence of one to three times a week. The decreased frequency of crying and unexplained and unsoothable crying episodes lasted 3 months after the intervention. The number of children whose parents perceived at least mild problematic crying reduced after the intervention. These findings are in line with previous research relating sleep problems with increased crying in young children [1, 5, 61, 62]. Several aspects could have caused these findings. First, in the sleep intervention, the parents learn to strengthen the child’s self-soothing competence, for example, by practicing falling and staying asleep more independently [43, 63]. Accordingly, the child relies less on parental co-regulation, which may reduce crying [5]. Second, the sleep intervention strengthened parental awareness and taught detailed soothing strategies [43, 64], which may help parents deal adequately with their children’s crying [65]. However, these findings must be interpreted with caution since no data on crying were available for the children who dropped out of the study. An unexpected finding was that crying due to defiance did not reduce after sleep intervention, although sleep problems were associated in previous research [15, 24, 66, 67]. It is conceivable that the effect was omitted in the present study because of drop-out bias, small sample size, or parent-reported assessment of crying due to defiance.

Across all measurement timepoints, the frequency of crying episodes decreased together with the reduction of nighttime awakenings. These results confirm and specify previous findings [22]. There is evidence that reduced nighttime awakenings lead to less fatigue in children, strengthening children’s self-regulatory capacity and may also reduce their crying [21]. Further, the present results indicate that across all measurement points the frequency of crying episodes decreased together with an increased nightly food intake. To clarify this result, we performed an additional item-level analysis, which showed that this was true only for crying in the morning (*β* = − 0.173 [− 0.278--0.069], *F* [1, 22.92] = 11.85, *p* = .002, *d* = 1.43). Since young children compensate for longer intervals without food at night with increased food intake in the morning [68], hunger could explain increased morning crying. Since sleeping through the night and getting out of the frequent nighttime feeding were issues with the parents [43], it may be unusual for the children to sleep longer without food. However, across all measurement time points unexplained and unsoothable crying decreased going along with higher sleep efficiency. Previous research found that children’s total sleep duration (an essential part of the sleep efficiency) is related to lower irritability and better self-soothing [15, 24, 69], suggesting that the child cries less unexplained and soothes more easily. Also crying due to defiance increased across all measurement time points together with lower sleep efficiency. This finding is in line with longitudinal research that postulated a significant correlation between lower sleep percent and behavioral regulation problems [70]. Further, crying due to defiance increased across all measurement time points together with less bed-sharing with the parents. Since sleeping in the child’s own bed is discussed in the intervention with the parents, this finding could indicate increased defiant behavior if the child was habituated to sleeping in the parent’s bed before the intervention and learns to sleep in their own bed after the intervention. However, surprisingly crying due to defiance decreased across all measurement time points together with more night awakenings. This result contradicts previous findings associated sleep problems with increased tantrums and defiance [15, 66, 67]. To clarify this finding future research should implement a larger sample and more objectifiable instruments, such as video analysis of an interaction during a clean-up task to assess defiant behavior [71] and acti- or polysomnography for sleep behavior.

The results indicated a reduction of child eating problems at 3 months after the intervention and an association with a decreased duration of nighttime awakenings. However, eating problems did not significantly reduce directly after the intervention. After 6 months, there was a significant reduction and the number of children whose parents perceived at least mild problematic eating patterns reduced after the intervention. However, these findings are difficult to interpret due to the high drop-out rate. Maybe the late improvement on the eating problem scale indicated no link to the sleep intervention. Also, it should be mentioned that in the studied sample, the eating problems at baseline were not very serious. Future studies should analyze a sample with more clinical eating problems to examine the effectiveness of the sleep intervention. However, the present results indicate that eating problems increased together with a longer duration of nighttime awakenings across all measurement points. This finding is consistent with previous research [72]. One possible explanation could be that children with eating difficulties often do not eat enough during the day and therefore awake during the night due to hunger or thirst [72]. The finding that eating problems decrease with progressing time may indicate that the eating problems in the present sample were more transient.

### Maternal and paternal distress may reduce after the sleep intervention

In the present study, the parental distress was reduced up to 6 months after the intervention and was associated with decreasing child nighttime awakenings, food intake, and unsoothable crying episodes. The maternal distress decreased significantly directly after the sleep intervention, but this reduction was no longer significant in the follow-up period. In contrast, paternal distress did reduce significantly at three and 6 months after the intervention. A possible explanation could be that maternal distress was reduced more in the sleep intervention because fathers were less involved in sleep-related parenting than mothers in the studied age group [73–75]. Furthermore, the results of the current study are consistent with previous research showing that behavioral sleep intervention for parents with sleep-disturbed children can lead beyond improvements in children’s sleep to reductions in parental distress [37, 76, 77]. Further, there were not many parents with high levels of parental distress in our sample. The fact that in our sample most parents were only moderately stressed could affect the interpretation of the study results. First, parental ratings of the child’s regulatory behavior might be influenced by the level of parental distress, as they might be perceived as less problematic when parents have lower to moderate levels of distress than when they have high levels of distress. Second, parents with only a moderate level of distress may have a lower willingness to engage in the intervention, which could contribute to the high levels of drop-out in the present study. Third, the study results could be biased if there is a tendency that highly stressed parents remained in the study, and parents with lower levels of distress may be no longer motivated to participate in the follow-up measures. And fourth, the interpretation of the present study results is limited to moderately stressed parents, and we have not yet been able to determine whether the intervention is effective in a sample with highly stressed parents. Evaluating effectiveness in highly stressed parents should be the next step, as in clinical practice high levels of parental distress are often associated with early child regulatory problems. However, the number of parents with high levels of distress reduced after the intervention, but the drop-out rate could also bias this.

The parental distress of mothers increased together with increased child’s nighttime awakenings across all measurement time points, which is in line with previous research [37]. In contrast, the fathers’ distress across all measurement points increased together with increased nighttime feeding of the child, increased unexplained and unsoothable crying, and reduced time progress. These differences are in line with previous research. It has been shown that fathers were less responsive at bedtime and night than mothers and had more parental tolerance for children’s nighttime crying [78, 79]. Also, it was assumed that fathers have fewer soothing strategies within their repertoire, which may lead to more difficulties soothing the crying child [80]. However, Dayton and colleagues [80] suggest a shift when mothers are weaning the child. Unfortunately, we do not know if the mother or the father did the nighttime feeding in the present study. However, it should be mentioned that differences may relate more to the distinction between the primary and secondary bedtime caregiver than between mothers and fathers [75]. Future research based on the present study should use separate measurement instruments for both parents to determine which sleep-related parenting tasks are performed by mothers and fathers and to what extent.

The drop-out rate in the present study was very high. However, our results indicated no difference between families who dropped out and families who stayed in the study in terms of demographic variables nor children’s sleep, crying, eating parameters, or parental distress. Parents who dropped out often mentioned reasons like a change in the family situation (e.g., birth, illness, divorce, a move, or job change). Further, it could be that families find it challenging to arrange a fixed appointment over 6 weeks. Subsequent studies could investigate whether drop-out rates can be reduced in a shorter intervention version. However, it is also conceivable that families were no longer motivated to complete the study materials if the sleep problem had improved or because it may have been too difficult for a parent to implement the intervention content alone at home. In both cases, personal contact over the follow-up period could potentially reduce the attrition rate. Because in this study families were contacted only by letter during the follow-up period, a recommendation for future studies would be to seek personal contact again at each measurement time point, for example, in a telephone interview or an additional face-to-face meeting and, if necessary, to provide multiple reminders for any outstanding parent study materials.

### Limitations

Several limitations need to be considered. First, a small sample with a substantial attrition rate was analyzed, resulting in some large differences between the analyzed groups. Since unequal sample sizes can lead to a loss of statistical power and an increased Type I error rate [81] we attempted to mitigate this with the use of Wilcoxon signed-rank tests that analyzed an individual’s repeated measure data as matched pairs which result in equal sample sizes [56]. For the explorative analyses, we choose the LMM method because it examines the within-subject level and is robust in cases of unbalanced data [57]. However, the results may not be representative and be influenced by sample selection bias. Second, any comparison to a control group is missing. Therefore, the current study does not provide a sufficient basis to interpret changes in sleeping, crying, and eating patterns and parental distress as a direct effect of Mini-KiSS sleep training. Hence, it is conceivable that the observed changes are related to other factors. The child’s sleep behavior over the study period could be influenced by the normal sleep development or child maturation which can have an impact on sleep behavior like teething [20, 28]. Third, only subjective and parent-reported data was used, and the crying and eating items were referred to a retrospective assessment. Also, there was no chance to diagnose clinically relevant eating problems in the present study. Future research should include diagnostic interviews at each measurement point in a sample with more clinically relevant crying and eating problems to shed light on a clinically relevant improvement of these regulatory problems after a sleep intervention. Fourth, due to the pilot nature of this study, the LMM analyses were run without *p*-value adjustment. In future research, a larger sample with a lower drop-out rate could be achieved through incentives or more telephone contact between follow-up periods. Future studies should include comparison with a control or waitlist control group and more advanced measurement methods, such as 24-hour logs or video analysis for crying and eating patterns and acti- or polysomnography for sleep behavior. However, this pilot study provides preliminary results suggesting that a parent-focused intervention to improve children’s sleep in individual settings may be a promising approach to reduce children’s sleep problems, crying, eating problems, and parental distress. Because early sleep problems are often associated with crying and eating problems and parental distress [5] clinicians might take away from these study results that a sleep intervention for sleep disturbed children may also have an impact on crying, and eating problems and parental distress, with reductions in the frequency and duration of nighttime awakenings and nighttime food intake and increases in sleep efficiency as the most related factors. The next steps are to validate the present findings in a randomized controlled trial including a larger sample of children affected by clinically relevant sleep, crying, and eating problems, and with parents who suffered from high levels of distress.

## Conclusions

Overall this single-arm pilot study suggests that a parent-focused intervention to improve child sleep may effectively impact child sleep problems, crying, eating problems as well as parental distress, with lasting effects between three and 12 months. Clinicians might take away from these study results that for young children with sleep disorders and comorbid crying problems, reducing the frequency of nighttime awakenings and associated increasing sleep efficiency might be relevant targets of intervention. To decrease comorbid eating problems, clinicians might focus on reducing the duration of nighttime awakenings. Among parents of young children with sleep disorders, clinicians could focus in their intervention on the frequency of the child’s nighttime awakenings as a related factor in reducing maternal distress and on the child’s nighttime feeding and unsoothable crying episodes in decreasing paternal distress.

A possible effect on clinically relevant crying and eating problems as on high levels of parental distress should be explored in subsequent studies. Further studies should implement a control group, a larger clinical sample, more personal contact during the follow-up period to reduce the attrition rate, and more reliable instruments such as 24-hour cry, eat and sleep logs, or video analysis to evaluate and refine these results.

## Supplementary Information

**Additional file 1: Table 1.** Descriptive statistics for outcomes at baseline across age groups, sexes, and sample attrition (drop-out after T1 vs. non-dropout). **Table 2.** Spearman correlations among the sleep variables for T1 (pre-intervention, *N*=59). **Table 3.** Spearman correlations among the sleep variables for T2 (post-intervention, *n*=36). **Table 4.** Spearman correlations among the sleep variables for T3 (three months after the intervention, *n*=25). **Table 5.** Spearman correlations among the sleep variables for T4 (six months after the intervention, *n*=18). **Table 6.** Spearman correlations among the sleep variables for T5 (twelve months after the intervention, *n*=14).

## Data Availability

The datasets generated and analyzed during the current study are available from the corresponding author on request.

## Abbreviations

BS: Bed-sharing
DEF: Crying due to defiance
DNW: Duration of nightly awakening
EAT: Difficulties in eating behavior
FNW: Frequency of nightly awakening
FRQ: Frequency of crying episodes
LMMs: Linear mixed models
Mini-KiSS: Mini-Kinderschlafstörungsprogramm (Mini Child Sleep Disorder Program)
NFI: Nightly food intake
PD-F: Parental distress of fathers
PD-M: Parental distress of mothers
PSI-SF: Parental Stress Index Short Form
SE: Sleep efficiency
SFS: Fragebogen zum Schreien, Füttern, Schlafen (questionnaire on crying, feeding, and sleeping)
SOL: Sleep onset latency
T1: Measurement point pre-intervention
T2: Measurement point post-intervention
T3: Measurement point 3 months after the intervention
T4: Measurement point 6 months after the intervention
T5: Measurement point 12 months after the intervention
TSD: Total nightly sleep duration
UUC: Unexplained and unsoothable crying
