# Supplementary material for: A single-arm pilot study: can a parental sleep intervention for sleep-disturbed young children in individual settings improve children’s sleep, crying, eating, and parental distress in mothers and fathers?
Source: BMC Pediatr. 2022 Oct 7;22:578. doi: 10.1186/s12887-022-03631-5 (PMC9541003; doi:10.1186/s12887-022-03631-5)
Supplement: Supplementary file 1 — Additional file 1: Table 1. Descriptive statistics for outcomes at baseline across age groups, sexes, and sample attrition (drop-out after T1 vs. non-dropout). Table 2. Spearman correlations among the sleep variables for T1 (pre-intervention, N=59). Table 3. Spearman correlations among the sleep variables for T2 (post-intervention, n=36). Table 4. Spearman correlations among the sleep variables for T3 (three months after the intervention, n=25). Table 5. Spearman correlations among the sleep variables for T4 (six months after the intervention, n=18). Table 6. Spearman correlations among the sleep variables for T5 (twelve months after the intervention, n=14). [file 12887_2022_3631_MOESM1_ESM.pdf]

Supplementary Material

Table 1. Descriptive statistics for outcomes at baseline across age groups, sexes, and sample attrition (drop-out after T1 vs. non-dropout)

	Initial age				Child's sex		Attrition	
	6-11 months	12-23 months	24-35 months	36-48 months	female	male	drop-out	non-dropout
	n=16	*n*=24	*n*=12	*n*=7	*n*=30	*n*=30	*n*=21	*n*=39
	Mnd (Q1-Q3)	*Mnd (Q1-Q3)*	*Mnd (Q1-Q3)*	*Mnd (Q1-Q3)*	*Mnd (Q1-Q3)*	*Mnd (Q1-Q3)*	*Mnd (Q1-Q3)*	*Mnd (Q1-Q3)*
SOL	20.22 (14.88-37.50)	26.43 (16.00-46.43)	30.35 (19.28-36.15)	18.57 (12.14-48.57)	26.22 (16.96-37.32)	26.43 (12.50-38.93)	31.43 (26.53-36.16)	19.29 (15.71-40.00)
FNW	4.00 (3.39-5.96)**	2.43 (1.43-3.57)**	1.28 (1.00-1.96)**	1.29 (0.86-1.43)**	2.22 (2.40-3.18)	2.86 (1.07-5.23)	1.93 (1.21-4.43)	2.43 (1.29-4.00)
DNW	37.50 (31.42-67.14)*	35.83 (16.43-55.71)*	8.93 (6.07-24.46)*	11.67 (7.14-17.14)*	23.93 (8.21-41.07)	30.71 (15.07-57.50)	29.64 (13.57-45.36)	26.43 (9.29-52.14)
TSD	620.14 (590.93-663.57)	614.29 (581.86-646.29)	639.50 (584.64-673.39)	625.00 (583.71-652.50)	622.50 (598.57-667.43)	601.40 (579.15-649.40)	600.36 (566.72-637.46)	621.86 (592.86-654.86)
SE	91.63 (83.32-93.33)	90.09 (86.89-92.11)	93.91 (89.72-96.07)	95.50 (88.69-96.04)	92.10 (88.50-94.89)	90.50 (86.99-93.49)	92.11 (87.21-94.31)	91.91 (88.11-94.77)
BS	1.00 (0.18-1.00)	0.57 (0.14-1.00)	0.64 (0.00-1.00)	0.42 (0.10-0.89)	0.72 (0.11-1.00)	0.57 (0.16-1.00)	0.86 (0.14-1.00)	0.50 (0.14-1.00)
NFI	1.00 (1.00-1.00)	0.86 (0.67-1.00)	0.86 (0.46-1.00)	0.21 (0.00-0.57)	0.86 (0.50-1.00)	0.86 (0.43-1.00)	1.00 (0.86-1.00)	0.80 (0.29-1.00)
FRQ	2.00 (1.25-2.69)	1.25 (1.00-1.94)	1.50 (1.06-2.25)	2.25 (1.00-2.50)	1.50 (1.00-2.06)	1.87 (1.19-2.50)	1.50 (1.25-2.13)	1.50 (1.00-2.25)
UUC	2.00 (2.00-3.00)	2.50 (1.50-3.00)	2.25 (2.00-2.87)	2.00 (2.00-2.50)	2.25 (1.50-3.00)	2.00 (2.00-3.00)	2.00 (1.50-3.00)	2.00 (2.00-2.50)
DEF	2.00 (1.25-3.00)	2.00 (1.00-2.75)	3.00 (2.00-3.00)	2.50 (2.00-3.25)	2.00 (1.50-3.00)	2.00 (2.00-3.00)	2.50 (2.00-3.00)	2.00 (1.75-3.00)
EAT	1.50 (1.17-1.69)	1.35 (1.09-1.88)	1.48 (1.27-1.73)	1.75 (1.31-2.38)	1.40 (1.15-1.69)	1.54 (1.29-1.92)	1.46 (1.27-1.69)	1.54 (1.15-1.77)
PD-M	31.00 (25.50-33.75)	27.00 (18.00-33.50)	29.00 (24.00-35.00)	26.00 (22.50-42.73)	28.00 (22.00-35.50)	29.00 (23.00-33.00)	29.23 (25.50-34.50)	28.00 (22.00-33.50)
PD-F	25.00 (19.64-29.75)	23.00 (17.00-29.00)	23.00 (17.50-30.50)	18.00 (15.50-26.50)	23.50 (17.75-30.50)	24.00 (17.77-28.00)	23.50 (19.00-32.00)	23.50 (17.00-28.50)

Notes: 'Drop-out' refers to participants who dropped out after T1. 'Non-dropout' refers to participants who completed at least T1 and one additional measurement point. Kruskal-Wallis tests were used to test differences among the age group conditions. Mann-Whitney *U* tests were used to test differences across outcomes for children's sex and sample attrition. For the differences in parental distress of mothers and fathers among children's age and sex, only parents with one child in the

study were analyzed. Therefore three parent pairs were excluded in these analyses because they had two siblings in the study. P-values are Benjamini-Hochberg adjusted. * $p < .05$, ** $p < .001$

Abbreviations: Mnd, median; Q1, 25th percentile; Q3, 75th percentile; SOL, sleep onset latency; FNW, frequency of nightly awakening; DNW, duration of nightly awakening; TSD, total nightly sleep duration; SE, sleep efficiency; BS, bed-sharing; NFI, nightly food intake; FRQ, crying frequency; UUC, unexplained and unsoothable crying; DEF, crying due to defiance; EAT, difficulties in eating behavior; PD-M, parental distress of mothers; PD-F, parental distress of fathers.

Table 2. Spearman correlations among the sleep variables for T1 (pre-intervention, *N*=59)

	SOL	FNW	DNW	TSD	SE	BS	NFI
SOL	-						
FNW	-.173	-					
DNW	-.057	.563***	-				
TSD	-.228	-.064	-.296	-			
SE	-.598***	-.287	-.695***	.466**	-		
BS	.134	.280	.150	.104	-.203	-	
NFI	-.020	.403**	.373*	-.189	-.266	.150	-

Note: *S*pearman correlations for the sleep parameters at T1 (pre intervention). P-values are adjusted according to Benjamini-Hochberg. * p < .05, ** p < .01, ***p < .001

Abbreviations: SOL, sleep onset latency; FNW, frequency of nightly awakening; DNW, duration of nightly awakening; TSD, total nightly sleep duration; SE, sleep efficiency; BS, bed-sharing; NFI, nightly food intake.

Table 3. Spearman correlations among the sleep variables for T2 (post-intervention, *n*=36)

	SOL	FNW	DNW	TSD	SE	BS	NFI
SOL	-						
FNW	.339	-					
DNW	.408*	.853***	-				
TSD	-.261	-.452*	-.546**	-			
SE	-.845***	-.613**	-.768***	.428*	-		
BS	.254	.330*	.425*	-.193	-.345	-	
NFI	.244	.593***	.655***	-.368	-.531**	-.026	-

Note: *S*pearman correlations for the sleep parameters at T2 (post intervention). P-values are adjusted according to Benjamini-Hochberg. * p < .05, ** p < .01, ***p < .001

Abbreviations: SOL, sleep onset latency; FNW, frequency of nightly awakening; DNW, duration of nightly awakening; TSD, total nightly sleep duration; SE, sleep efficiency; BS, bed-sharing; NFI, nightly food intake.

Table 4. Spearman correlations among the sleep variables for T3 (three months after the intervention, *n*=25)

	SOL	FNW	DNW	TSD	SE	BS	NFI
SOL	-						
FNW	.020	-					
DNW	.075	.874***	-				
TSD	-.374	-.223	-.106	-			
SE	-.723***	-.530*	-.607**	.358	-		
BS	.220	.208	.388	.256	-.210	-	
NFI	.164	.608**	.771***	-.279	-.518*	.362	-

Note: *S*pearman correlations for the sleep parameters at T3 (three months after intervention). P-values are adjusted according to Benjamini-Hochberg. * p < .05, ** p < .01, ***p < .001

Abbreviations: SOL, sleep onset latency; FNW, frequency of nightly awakening; DNW, duration of nightly awakening; TSD, total nightly sleep duration; SE, sleep efficiency; BS, bed-sharing; NFI, nightly food intake.

Table 5. Spearman correlations among the sleep variables for T4 (six months after the intervention, *n*=18)

	SOL	FNW	DNW	TSD	SE	BS	NFI
SOL	-						
FNW	.218	-					
DNW	.084	.909***	-				
TSD	-.383	-.633*	-.545*	-			
SE	-.603*	-.688**	-.708**	.492	-		
BS	-.040	.430	.353	-.244	-.099	-	
NFI	-.018	.735**	.845***	-.490	-.579*	.384	-

Note: *S*pearman correlations for the sleep parameters at T4 (six months after intervention). P-values are adjusted according to Benjamini-Hochberg. * p < .05, ** p < .01, ***p < .001

Abbreviations: SOL, sleep onset latency; FNW, frequency of nightly awakening; DNW, duration of nightly awakening; TSD, total nightly sleep duration; SE, sleep efficiency; BS, bed-sharing; NFI, nightly food intake.

Table 6. Spearman correlations among the sleep variables for T5 (twelve months after the intervention, *n*=14)

	SOL	FNW	DNW	TSD	SE	BS	NFI
SOL	-						
FNW	.166	-					
DNW	-.193	.398	-				
TSD	-.347	-.597	-.267	-			
SE	-.878***	-.337	-.237	.484	-		
BS	.295	.686	.117	-.123	-.297	-	
NFI	.086	.396	.425	-.371	-.226	.270	-

Note: *S*pearman correlations for the sleep parameters at T5 (twelve months after intervention). P-values are adjusted according to Benjamini-Hochberg. ***p < .001

Abbreviations: SOL, sleep onset latency; FNW, frequency of nightly awakening; DNW, duration of nightly awakening; TSD, total nightly sleep duration; SE, sleep efficiency; BS, bed-sharing; NFI, nightly food intake.
